# Transfer of motion through a microelectromechanical linkage at nanometer and microradian scales

**DOI:** 10.1038/micronano.2016.55

**Published:** 2016-09-12

**Authors:** Craig R. Copeland, Craig D. McGray, Jon Geist, Vladimir A. Aksyuk, Samuel M. Stavis

**Affiliations:** 1 Center for Nanoscale Science and Technology, National Institute of Standards and Technology, Gaithersburg, MD 20899, USA; 2 Maryland Nanocenter, University of Maryland, College Park, MD 20742, USA; 3 Engineering Physics Division, National Institute of Standards and Technology, Gaithersburg, MD 20899, USA; 4 Modern Microsystems, Silver Spring, MD 20904, USA

**Keywords:** Electrothermal, linkage, microelectromechanical, nanoparticle, noise, rotation, tracking, translation

## Abstract

Mechanical linkages are fundamentally important for the transfer of motion through assemblies of parts to perform work. Whereas their behavior in macroscale systems is well understood, there are open questions regarding the performance and reliability of linkages with moving parts in contact within microscale systems. Measurement challenges impede experimental studies to answer such questions. In this study, we develop a novel combination of optical microscopy methods that enable the first quantitative measurements at nanometer and microradian scales of the transfer of motion through a microelectromechanical linkage. We track surface features and fluorescent nanoparticles as optical indicators of the motion of the underlying parts of the microsystem. Empirical models allow precise characterization of the electrothermal actuation of the linkage. The transfer of motion between translating and rotating links can be nearly ideal, depending on the operating conditions. The coupling and decoupling of the links agree with an ideal kinematic model to within approximately 5%, and the rotational output is perfectly repeatable to within approximately 20 microradians. However, stiction can result in nonideal kinematics, and input noise on the scale of a few millivolts produces an asymmetric interaction of electrical noise and mechanical play that results in the nondeterministic transfer of motion. Our study establishes a new approach towards testing the performance and reliability of the transfer of motion through assemblies of microscale parts, opening the door to future studies of complex microsystems.

## Introduction

Control over the output of motion is an essential function of many mechanical systems, so the deterministic transfer of motion through assemblies of parts is of fundamental importance. Mechanisms with moving parts in contact are ubiquitous for this purpose at the macroscale. In principle, the utility of such mechanisms extends down to smaller length scales. In practice, challenges involving the operation^1–3^ and characterization of such mechanisms limit the many potential applications of this technology at the microscale, restricting the functionality of microsystems. These challenges of microsystem technology and motion metrology are at least correlated. As a result of these challenges, modern microsystems commonly incorporate compliant mechanisms^4,5^ that eliminate contact between moving parts, but this trend in microsystem design has proceeded without a complete understanding of the limits of operation of rigid-link mechanisms.

For example, engineers have demonstrated linkages for microsystems^6–9^, but have not investigated the related limits of performance and reliability at the smallest relevant scales. There are at least three challenges to such investigations. One of the most basic functions of a mechanical linkage, at any scale, is to convert linear motion to rotary motion, so the first challenge is to perform quantitative measurements at relevant length and angle scales. Many microsystems implement linear motion at nanometer scales^10–15^ and a variety of optical methods can measure this mode of motion^16–21^. Rotation at the microradian scale is a less common mode of motion of some microsystems^22–27^ that is more difficult to measure. The second challenge is to characterize the kinematics of microsystems in operation, which requires simultaneous measurement of the motion of multiple parts in an assembly. This is because coupling interactions such as play and stiction can affect the kinematics of the microsystem, often in ways that are difficult to control and predict because of fabrication tolerances and surface forces^1–3^. The third challenge is to resolve single cycles of motion, as opposed to a mean value of motion over multiple cycles. This is particularly important in cases when coupling interactions result in motion that is aperiodic or nondeterministic. In any case, single motion cycles are essential for a variety of applications in which the motion of the microsystem is fundamentally aperiodic, such as positioning and switching^6,10,28^.

**Figure 1:**
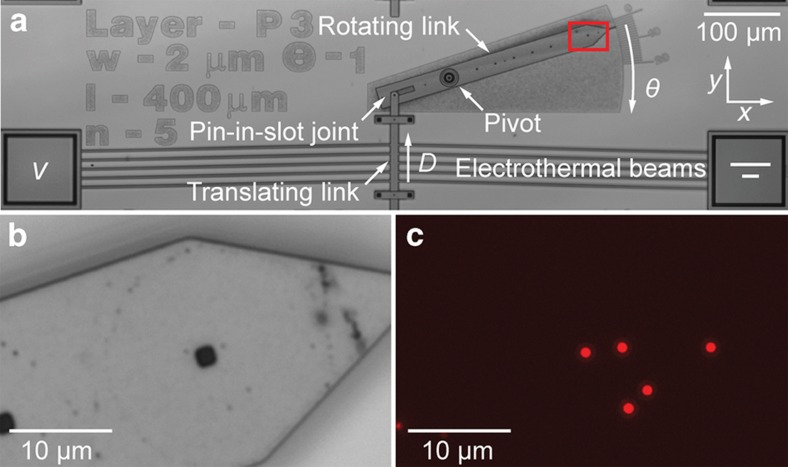
Microsystem for testing the transfer of motion through an assembly of parts at microradian and nanometer scales. (**a**) Bright-field micrograph showing a microelectromechanical linkage with a translating link coupling through a pin-in-slot joint to a rotating link on a pivot. The red rectangle denotes the region of interest in (**b** and **c**). (**b**) Bright-field micrograph showing the region of interest. A dark etch hole ≈2 μm in diameter is visible near the center of the image. (**c**) Fluorescence micrograph showing fluorescent nanoparticles labeling the same region of interest. Changes in the position of the centroid and the orientation of either the etch holes or the fluorescent nanoparticles indicate the underlying motion of the rotating link.

To meet these challenges, we develop a novel combination of optical microscopy methods to track surface features and fluorescent nanoparticles that indicate the motion of the underlying parts of a microsystem. These measurement methods enable the first quantitative study of the transfer of motion through a microelectromechanical linkage at nanometer and microradian scales. Figure 1 shows the microsystem, which integrates a translating link coupling through a pin-in-slot joint to a rotating link on an eccentric pivot^6^. This test system is representative of a class of microsystems that transfer motion through assemblies of parts to perform work requiring complex kinematics. Sandia National Laboratories originally designed such microsystems for extremely consequential applications in positioning and switching^29,30^, and numerous applications of complex microsystems ranging from microrobotics^13^ to microsurgery^31^ are emerging. Despite the conceptually simple design of the test system, our measurements reveal that this assembly of microscale parts exhibits complex motions that are below the optical resolution limit, outside the scope of typical models for microelectromechanical systems and critical to the performance and reliability of microsystems for practical applications. In this way, our new approach to measuring the transfer of motion through assemblies of microscale parts opens the door to the future study of more complex microsystems, which is necessary to realize their full potential.

## Materials and methods

### Microelectromechanical system

Our test system is a microelectromechanical linkage fabricated in polysilicon by an ultraplanar multilevel process^32^. The translating link is the shuttle of a V-beam electrothermal actuator^8,33^, consisting of five compliant beams with common electrical connections at the fixed ends, and a nominally rigid bisecting beam. An input voltage *v* causes an electrical current to flow through the compliant beams, which reversibly heat and expand. The magnitude of heating and beam expansion depends on the magnitude of *v*. The V-shape of the beams and two guides nominally confine this motion to the substrate plane, translating the shuttle. A pin-in-slot joint connects this translating link to one end of the rotating link. The joint pin is fixed to the translating link and passes through a slot in the rotating link. The translation of the translating link *D* nominally results in rotation of the rotating link *θ* around a pivot. A clearance of several micrometers between the linkage pin and slot edges results in play in the joint, referring to the maximum distance *D*_play_ or angle *θ*_play_ through which the translating link or the rotating link moves without applying appreciable force or motion to the other link. Play in the pivot refers to clearance between the rotating link and the pivot.

We actuate the linkage with two protocols. A staircase function input voltage moves the linkage through a single motion cycle in many steps over a large fraction of its operable range. We use this first protocol to characterize the kinematics of the linkage. A square wave input voltage moves the linkage through many motion cycles in single steps between *low* and *high* values. We use this second protocol to test the repeatability of the rotational output at several points in the operable range. For this second protocol, we measure the input voltages at 8 Hz and actuate the linkage at the Nyquist frequency of 4 Hz. The Supplementary Methods and Supplementary Figure S1 present details of these protocols.

### Microscopy methods

We combine two methods, based on bright-field microscopy and fluorescence microscopy, to track surface features and fluorescent nanoparticles as indicators of the motion of the translating and rotating links. Each method has advantages and disadvantages, and our novel combination of the two leverages the advantages of both. We use the same microscope system for both methods, recording optical micrographs with a pixel size of 127.2 nm±0.1 nm. The latter value denotes a limit of uncertainty, based on Fourier analysis of optical micrographs of periodic features with a known pitch^34^. Supplementary Figure S2 shows this calibration and the Supplementary Methods present further details of the microscope system. We subsequently report each quantity as a mean value and each uncertainty as a standard deviation (s.d.), or we note otherwise.

To characterize the transfer of motion through the linkage from input to output, we use bright-field microscopy and track native surface features to simultaneously measure the motion of both links. Many bright-field methods^17,18,21^ for tracking surface features, typically edges, measure only translational motion in one dimension. In contrast, we localize surface features to measure motion in two dimensions with three degrees of freedom and to characterize the linkage geometry. The change in the position of the linkage pin that passes through the translating link indicates *D*, and the rotation of the line of etch holes on the rotating link indicates *θ*. Figure 1b shows one of the etch holes on the rotating link. The position of the pivot, which Figure 1a shows, serves as a reference to correct for microscope drift. These surface features appear as dark spots in bright-field micrographs due to the reduced intensity of reflected light relative to the surrounding surface. We convert bright-field micrographs to binary images by thresholding and localize each surface feature by centroid analysis. Measurement uncertainties range from 9 nm to 12 nm for measurements of *D* and from 90 μrad to 140 μrad for measurements of *θ*. Supplementary Note 1 presents details of the uncertainty evaluation. This tracking method has the advantages of requiring no modification of the linkage and of resolving its motion and geometry at scales that are far smaller than the optical diffraction limit. However, this method requires the presence of surface features on specific parts of the microsystem and that the features provide sufficient contrast under bright-field illumination, which depends on the design and fabrication of the microsystem.

To characterize the repeatability of the rotational output of the linkage, we deposit a microscale constellation of fluorescent nanoparticles on the rotating link, image the nanoparticles by fluorescence microscopy and track the positions of the nanoparticles during actuation^35–37^. The rotation of the constellation of fluorescent nanoparticles indicates *θ*. Figure 1c shows a subset of this constellation on the rotating link. The image of each isolated particle appears as the point spread function of the microscope system. We localize the particles by fitting the image of each particle to a bivariate Gaussian function. We use the method of damped least squares to perform all fits in this study. The measurement uncertainty of *θ*, which varies slightly between experiments, ranges from 16 μrad to 23 μrad. Supplementary Note 3 presents details of this uncertainty evaluation, which is comprehensive. This measurement method has several advantages. Fluorescent nanoparticles emit signals that are spectrally distinct from surface features in bright-field micrographs, as Figures 1b and 1c show, providing contrast that is largely independent of the underlying microsystem. Similarly, the number of fluorescent nanoparticles is largely independent of the design of the microsystem, being limited primarily by its surface area and in our experiment exceeding the number of etch holes by a factor of two. Importantly, this tracking method builds on the firm theoretical foundation of localizing point sources^37–40^, enabling novel evaluations of uncertainty and guiding future work. Experimental uncertainties are larger than the theoretical minimum uncertainties, which the number of detected photons and constellation size determine^37–39^, by a factor of only 1.3 to 1.6, indicating a nearly ideal measurement. Analysis of Allan deviations^41^ shows the absence of drift in measurements of *θ*, demonstrating an approach towards reducing uncertainty below 1 μrad by temporal averaging. The future use of a constellation of a larger number of brighter particles will enable even better spatial precision and temporal bandwidth. Analysis of the rigidity of the point cloud between images reveals unintended motion of the rotating link out of the image plane, deforming the image of the nanoparticle constellation. This deformation produces a negligible bias in the measured planar rotation that we nonetheless correct to ensure accuracy. The future development of the measurement method to analyze the deformation of the image of the nanoparticle constellation will enable tracking of motion out of the image plane. The use of fluorescent particles as optical indicators is not always possible, however, as is the case with the translating link, which becomes sufficiently hot during operation to destroy the fluorescent dye in the nanoparticles. The use of nanoparticle indicators that can withstand high temperatures will be the subject of a future study.

Both methods of optical microscopy and image analysis produce a point cloud of positions in the image plane corresponding to either surface features or fluorescent nanoparticles, (*x*_*j,η*_,*y*_*j,η*_), where the index *j* denotes an image in a measurement series and the index *η* denotes a point in a point cloud. We determine the planar kinematics of the underlying link by applying a rigid transformation to map the point cloud in image *j* to the point cloud in image *k.* This transformation consists of a displacement of the centroid of the point cloud of magnitude $D=\sqrt{{\left(X_{j}-X_{k}\right)}^{2}+{\left(Y_{j}-Y_{k}\right)}^{2}}$, and a rotation of the point cloud about the centroid, $\Delta \theta =\left|\theta_{j}-\theta_{k}\right|$, where (*X*_*j*_,*Y*_*j*_) and (*X*_*k*_,*Y*_*k*_) are the positions of the centroids in images *j* and *k*, respectively, and *θ*_*j*_ and *θ*_*k*_ are the orientations of the point cloud in images *j* and *k*, respectively. The optimal rigid transformation minimizes the root-mean-square distance, or error, between corresponding points in images *j* and *k.* In the case of the translating link, which has only a single surface feature for tracking, changes in the position of the single point give the displacements.

## Experiments and results

### Linkage motion

Figure 1a shows the linkage in a nominally static condition in the absence of an input voltage. The normal operation and basic kinematics of the linkage are as follows. As input voltage *v* increases from 0 V, the translating link advances forward through the play in the joint $D_{\mathrm{play}}^{0}$, but does not actuate the rotating link. At a minimum voltage for actuation *v*_min_, the translating link couples to the rotating link and rotates it forward by sliding contact. As *v* decreases, the translating link decouples from the rotating link and retracts back through the play in the joint. As *v* decreases further, the translating link recouples to the rotating link and rotates it backward by sliding contact, completing a motion cycle. The response time of the electrothermal actuator is on the order of 1 ms (Ref. 42), which is much faster than the timing of any of the measurements. Accordingly, motion blur from actuation is absent from all optical micrographs, indicating that the linkage is nominally stationary during micrograph acquisition.

**Figure 2:**
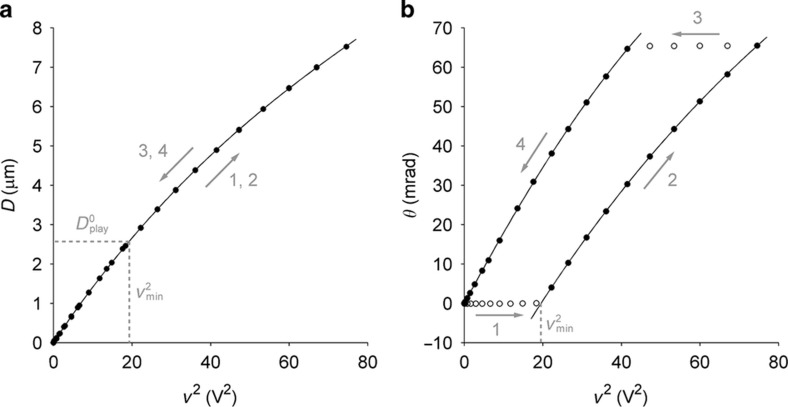
Measurement and analysis of the electrothermal actuation of the linkage. (**a**) Scatterplot showing that a third-order polynomial empirically models the displacement of the translating link *D* in response to the square of the voltage applied to the electrothermal actuator *v*^2^. Dashed gray lines indicate electromechanical characteristics of the joint pertaining to play. (**b**) Scatterplot showing that a piecewise quadratic function empirically models the rotation of the rotating link *θ* in response to *v*^2^. Play in the joint results in hysteretic motion of the rotating link. The horizontal-axis intercept of the response function for section 2 of the hysteresis loop gives *v*_min_. For an input of this value, the model in (**a**) outputs $D_{\mathrm{play}}^{0}$. Uncertainties are smaller than the data markers.

V-beam electrothermal actuators require complex models coupling electrical, thermal and mechanical effects for a physical description of the actuator response^43^. Such numerical models are generally consistent with experimental measurements within an accuracy of 1% to 10% (Refs. 30,43,44,45,46). The further development of such models to improve their predictive accuracy is not the purpose of our study. Rather, we use an empirical analysis to accurately model the experimental response that we precisely measure during operation of the linkage. Figures 2a and 2b show our measurement results and analysis. A third-order polynomial accurately models *D* in response to the square of the voltage applied to the electrothermal actuator *v*^2^ over the experimental range tested, resulting in an adjusted *R*^2^ value of 0.99999. The coupling and decoupling of the links caused by the play in the joint produces hysteresis in the response of *θ* to *v*^2^ over a single motion cycle, as Figure 2b shows. A piecewise quadratic function accurately models this response, resulting in an adjusted *R*^2^ value of 0.99997 for section 2 of the hysteresis loop and 0.99995 for section 4. This empirical analysis provides two important characteristics of the actuation of the linkage. The horizontal-axis intercept of the empirical model for section 2 of the response of *θ* to *v*^2^ gives *v*_min_=4.40 V±0.03 V. For this value of *v*_min_ as an input, the empirical model in Figure 2a outputs a value of $D_{\mathrm{play}}^{0}$=2 588 nm±35 nm. The relative uncertainty of *v*_min_ is an order of magnitude smaller than the corresponding uncertainties of existing models and measurements of electrothermal actuation, emphasizing the precision of the measurement method.

**Figure 3:**
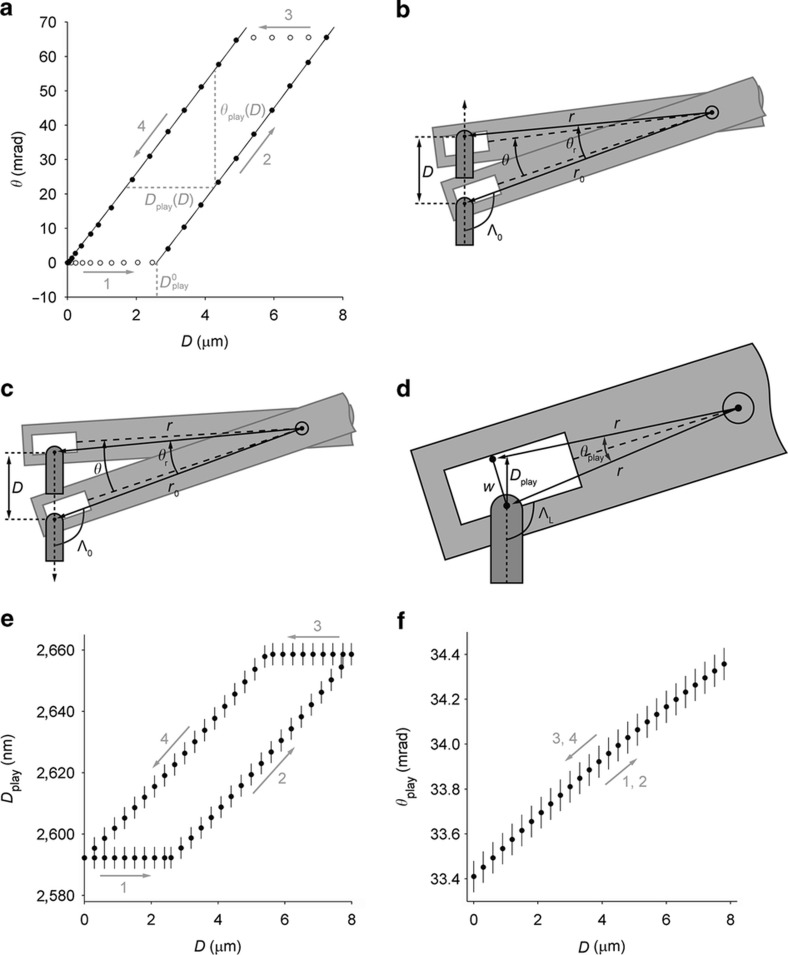
Measurement and analysis of the mechanical kinematics of the linkage. (**a**) Scatterplot showing a direct comparison of the motion of the translating and rotating links, isolating the kinematics of the linkage. The black line in section 2 is a fit to Equation (2), determining the value of $D_{\mathrm{play}}^{0}$. The black line in section 4 is a plot of Equation (4). The uncertainties are smaller than the data markers. (**b**) Schematic showing the linkage kinematics for sections 1 and 2, and Equation (2). (**c**) Schematic showing the linkage kinematics for section 4 and Equation (4). (**d**) Schematic showing the maximum angular play in the rotation of the rotating link while the links are decoupled. (**e**) Scatterplot showing the predicted values of *D*_play_(*D*) based on the determined value of $D_{\mathrm{play}}^{0}$. (**f**) Scatterplot showing the predicted values of *θ*_play_(*D*) based on the determined value of $D_{\mathrm{play}}^{0}$. The vertical bars in (**e** and **f**) are 1 s.d.

The transfer of motion through the linkage does not depend on the specific mechanism of electrothermal actuation. Figure 3a directly compares the motion of the translating and rotating links, isolating their kinematics for study. We develop a kinematic model to predict the transfer of motion through this assembly of parts for comparison with our measurement results. Although methods exist for modeling the output motion and associated error of complex linkages^47^, the linkage in our study has only two links, so geometric relationships and uncertainty propagation suffice to model the output motion and associated uncertainty. In a novel analysis, our kinematic model uses geometric parameters of the linkage that we independently obtain by precisely localizing its surface features. This ensures the accuracy of these parameters with respect to the experimental microsystem, rather than using nominal values from the design of the linkage that do not account for fabrication tolerances. Figures 3b–d show these parameters. From this analysis, we obtain *r*_0_=74 073 nm±7 nm as the magnitude of the vector $\vec{r}$ between the centroids of the pivot and the linkage pin at *D*=0, Λ_0_=1.8866 rad±0.0005 rad as the angle between $\vec{r}$ and the trajectory of the translating link at *D*=0 and Λ_L_=1.8731 rad±0.0014 rad as the angle between the links at *D*=0. Supplementary Figure S3 presents the details of the superresolution measurement of Λ_0_ and Λ_L_. Our kinematic model assumes that the trajectory of the translating link is straight, and that there is no play in the pivot, so the center of rotation is coincident with the center of the pivot. We revisit these simplifying assumptions in the following comparison of measured and predicted motion, which reveals subtle deviations from ideal motion at nanometer and microradian scales, and thus elucidates the experimental motion of the microsystem.

The kinematic model consists of four equations corresponding to the four sections of the hysteresis loop that Figure 3a shows. Sections 1 and 2 of Figure 3a correspond to an advancing translating link:

(1)θ+(D)=0,D⩽Dplay0

(2)θ+(D)=θr→(D)−12(θplay(0)+θplay(D)),Dplay0⩽D<Dmax

Sections 3 and 4 of Figure 3a correspond to a retracting translating link:

(3)θ−(D)=θ+(Dmax),Dmax−Dplay(Dmax)⩽D⩽Dmax

(4)θ−(D)=θr→(D)−12(θplay(0)−θplay(D)),D<Dmax−Dplay(Dmax)

Figure 3b illustrates Equation (2) and Figure 3c illustrates Equation (4). The maximum input voltage sets the maximum value of *D*, *D*_max_.

The function $\theta_{\vec{r}}\left(D\right)$ is the rotation of the vector $\vec{r}$ between the pivot and the linkage pin, which models the hypothetical kinematics of the linkage for sliding contact along the center line of the rotating link, in the absence of play in the joint:

(5)θr→(D)=12(Λ0−Real[i⋅ln(r0+DeiΛ0D+r0eiΛ0)])

Figures 3b and 3c illustrate this rotation and Supplementary Note 2 describes the details of the derivation. The function *θ*_play_(*D*), which describes the maximum possible rotation of the rotating link when it is decoupled from the translating link for a given value of *D*, modifies $\theta_{\vec{r}}\left(D\right)$ to account for the presence of play in the joint. From the schematic of this decoupled state that Figure 3d shows, the law of cosines gives:

(6)θplay(D)=cos−1(1−w22⋅r(D)2)

where $w=D_{\mathrm{play}}^{0}\sin\left(\pi -\Lambda_{L}\right)$ is the effective width of the slot after accounting for the diameter of the pin. Supplementary Note 2 describes the derivation of *r*(*D*), which follows from Figures 3b and 3c. Figure 3a illustrates *θ*_play_(*D*) as a vertical dashed gray line. The function *D*_play_(*D*) is the linear play in the joint, determining the hysteresis that Equations (3) and (4) describe:

(7)Dplay(D)=wsin(π−ΛL−θ(D))

Figure 3a illustrates *D*_play_(*D*) as a horizontal dashed gray line. Figure 3e shows a plot of *D*_play_(*D*), which has a hysteretic response because of the dependence on *θ*(*D*).

The black line in section 2 of Figure 3a is a fit to Equation (2) with $D_{\mathrm{play}}^{0}$ as the only adjustable parameter, resulting in an adjusted *R*^2^ value of 0.99996. This analysis gives $D_{\mathrm{play}}^{0}$=2 592 nm±4 nm, which is in quantitative agreement with the value from the empirical analysis of electrothermal actuation that Figure 2 shows. This is an important result, confirming the consistency of a kinematic model describing the motion of the links as an assembly of interacting parts, and an empirical model describing the electrothermal actuation of each link independently. This consistency builds confidence in the quantity $D_{\mathrm{play}}^{0}$, which is central to the transfer of motion through the linkage, and for which this study demonstrates a novel method for precise measurement *in operando*. The relative uncertainty of the value of $D_{\mathrm{play}}^{0}$ from the kinematic analysis, which directly relates *θ* and *D*, is one order of magnitude smaller than that of the empirical analysis of electrothermal actuation, which requires two models with associated uncertainties to relate *θ* and *D*. The black line in section 4 of Figure 3a is a plot of Equation (4) using this value of $D_{\mathrm{play}}^{0}$.

In addition, we use this value of $D_{\mathrm{play}}^{0}$ to determine *θ*_play_(*D*), as Figure 3f shows. The angular play in the joint is another important characteristic of the linkage—not only for an accurate description of the kinematics, but also for informing of how feedback can affect the rotational output in applications incorporating the linkage within a more complex microsystem. For example, if the rotational output of the linkage was to interact with another part of a microsystem, then the forces acting on the rotating link through the coupling to that part could potentially change the orientation of the link by $\delta \theta \;⩽\;\theta_{\mathrm{play}}\left(D\right)$.

The ability to predict *θ*(*D*) with no more than one adjustable parameter allows for a meaningful analysis of how the measured kinematics of the linkage deviate from the ideal model during operation. The use of additional adjustable parameters for *r*_0_, Λ_0_ and Λ_L_ would lump the details of any such deviations into the fitted values, emphasizing the utility of superresolving and fixing these parameters. We find that the measured kinematics are in good agreement with the ideal model, as Figure 3a shows. However, Supplementary Figure S4 shows that systematic deviations from the model become significant beyond a confidence interval of 95%. These deviations, which have a mean value of ≈5% of the measured values of *θ*(*D*), result from experimental kinematics that the ideal model does not describe.

One such nonideality, which Supplementary Figure S3 shows, is that the trajectory of the translating link is not perfectly linear, as the kinematic model assumes, which is evidently due to some heterogeneity of the electrothermal beams. Supplementary Figure S5 shows how this nonideality produces deviations between the predicted and measured values of *r*(*D*). However, these residuals fall within uncertainty to 95% confidence and do not correlate with the deviations between predicted and measured values of *θ*(*D*). Nonetheless, this result suggests the utility of our methods for measuring the performance and reliability of compliant mechanisms, which are sensitive to material and dimensional properties that vary within fabrication tolerance^4,5^.

Another nonideality is play in the pivot, allowing the point of rotation to change during operation in a way that is difficult to predict, causing the measured kinematics to deviate slightly from the ideal model. We can empirically account for this nonideality by adjusting the value of *r* in Equations (S2) and (6) to modify the position of the point of rotation relative to the linkage pin. Supplementary Figure S4 shows that changes in the point of rotation over a range of ≈200 nm can explain these deviations. These results emphasize the utility of the measurement method for revealing how the practical limitations of the fabrication and operation of assemblies of microscale parts affect their kinematics.

**Figure 4:**
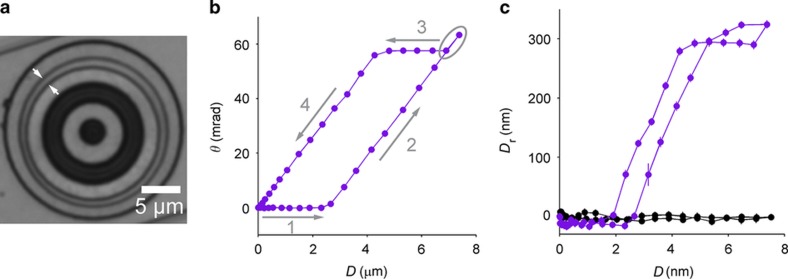
The atypical presence of stiction alters the kinematics of the linkage. (**a**) Bright-field micrograph showing the pivot with white arrows indicating the play. (**b**) Scatterplot showing that stiction maintains the coupling between the links, altering the hysteretic motion. (**c**) Scatterplot showing that stiction causes radial translation of the rotating link with respect to the pivot *D*_*r*_ in response to the translation of the translating link. Stiction between the links produces a force acting parallel to the rotating link that is sufficient to push and pull the rotating link through the play in the pivot over a single motion cycle (purple). In the typical absence of stiction, the rotating link does not translate (black). The vertical bars are 1 s.d. and are typically smaller than the data markers.

Play in the pivot, which during normal operation leads to a mean deviation from ideal rotation of ≈5%, becomes more significant in the atypical presence of transient stiction in the joint. The white arrows in Figure 4a indicate this play. Exposure of the microsystem to ambient conditions for a duration of multiple weeks alters the intrinsic properties of the contacting surfaces. The resulting stiction increases the coupling force between the links, affecting the motion of the rotating link in two ways. First, because of the increase in the component of the coupling force that acts perpendicular to the rotating link, the links do not immediately decouple in response to a decrease in *v*^2^, altering the hysteresis in the response of the rotating link. The gray ellipse in Figure 4b indicates this delayed decoupling. Second, independently of the rotation, the component of the coupling force that is parallel to the rotating link becomes sufficiently large to push and pull the rotating link through the play in the pivot over a single motion cycle. Figure 4c shows these results. From the range of this motion, we measure the effective play in the pivot as 342 nm±9 nm. This is in contrast to normal operation in the absence of stiction, in which the parallel component of the coupling force is too small to produce any significant motion of the rotating link in the radial direction. We restore the normal operation of the linkage by actuating it through several motion cycles, after which the effects of stiction are no longer apparent in the measured kinematics. These results are relevant to the storage and operation of microsystems with moving parts in contact in ambient conditions, and motivate further tests of the performance and reliability of the linkage.

### Linkage repeatability

The repeatability of the rotational output is a primary metric of the performance and reliability of the linkage. Fluctuations in measured rotational output can result from a combination of factors, including baseline variation due to measurement uncertainty, intrinsic variation of the material or surface properties of the microsystem, and extrinsic variation in input voltage and environmental factors. We measure the repeatability of the rotational output over thousands of motion cycles by tracking fluorescent nanoparticles on the rotating link. Our quantitative evaluation of measurement uncertainty and the empirical model of electrothermal actuation allow us to discriminate among these factors and characterize the repeatability of the rotational output.

**Figure 5:**
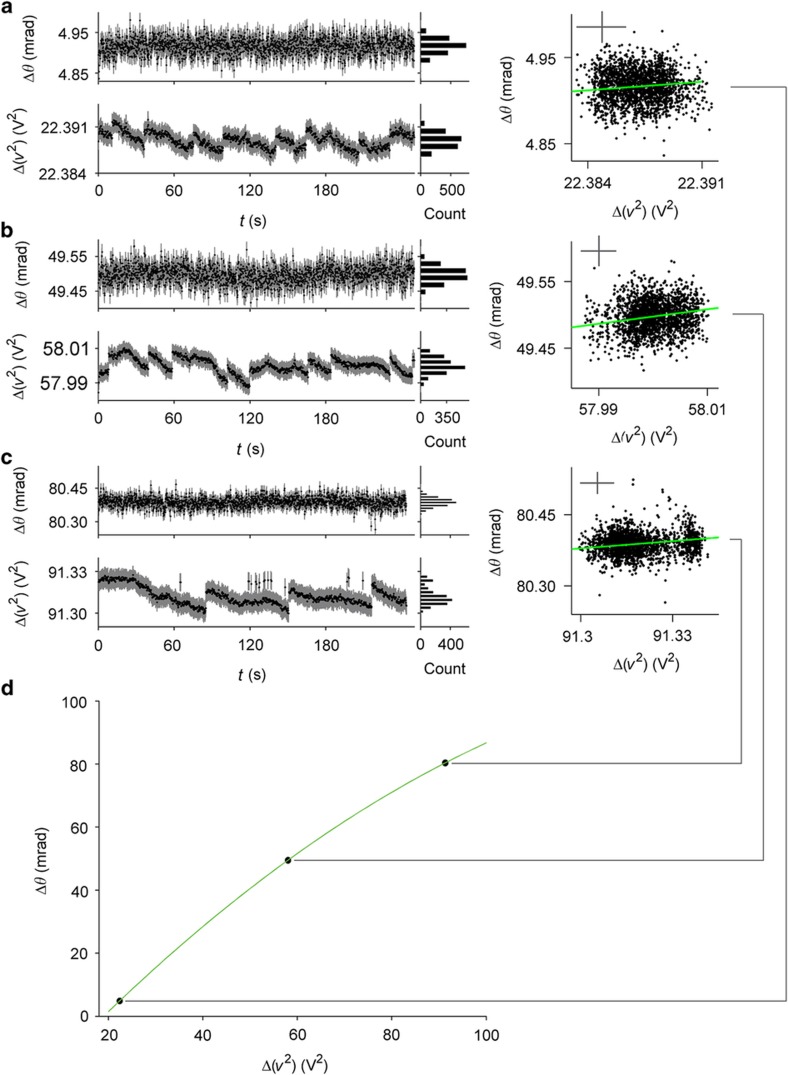
Input voltages with submillivolt noise result in a deterministic output. (**a–c**) Measurement series, histograms and scatterplots showing the local response of Δ*θ* to changes in Δ(*v*^2^) at nominal voltage values of 4.5 V, 7.5 V and 9.5 V, respectively. Input voltages with submillivolt noise result in rotational outputs that are deterministic within measurement uncertainty. (**d**) Scatterplot showing the global response of Δ*θ* to changes in Δ(*v*^2^) with a fit to the response function as a green line. The vertical bars for values of Δ*θ* are empirical uncertainties that the main text describes. The vertical bars for values of Δ(*v*^2^) are uncertainties that the instrument manufacturer specifies. The small scatterplots in (**a**–**c**) show representative uncertainties in the upper left corners.

We first test the intrinsic stability of the microsystem by repeatedly actuating the linkage using input voltages with electrical noise on the scale of hundreds of microvolts, which is the lowest that we can source. The Supplementary Methods and Supplementary Figure S1 present details of this actuation protocol. The following analysis considers the magnitude of changes in *θ* and *v*^2^ between sequential *low*-to-*high* and *high*-to-*low* transitions, Δ*θ* and Δ(*v*^2^). Square wave amplitudes, Δ*v*, of the three levels of input voltage and associated values of root-mean-square noise are 4.5650 V and 0.0003 V, 7.5646 V and 0.0005 V, and 9.4573 V and 0.0006 V, respectively. Figures 5a–c show the corresponding values of Δ(*v*^2^) and Supplementary Figures S10a,c and e show the power spectral densities, which reveal a frequency dependence that is consistent with pink noise. At these three levels of input voltage, submillivolt noise results in repeatable motion of the linkage over 2000 transitions. Supplementary Table S2 shows that the standard deviations of the rotational output for the three levels of input voltage are equal to the measurement uncertainties of 18 μrad, 23 μrad and 23 μrad. This consistency of the rotational output demonstrates the deterministic transfer of motion through the linkage over the entire measurement series. This indicates the aggregate invariance of the intrinsic properties of the microsystem, including the electrothermal actuator during heat cycling and the mechanical linkage with moving parts in contact, within measurement uncertainty. The simplest explanation for this result is that the intrinsic properties of the microsystem are individually invariant. Any effects of submillivolt noise and environmental fluctuations are also within the measurement uncertainty. Future work will reduce the measurement uncertainty to further investigate all of these factors. Figure 5d shows Δ*θ* in response to actuation at the three levels of voltage plotted as a function of Δ(*v*^2^) with a global fit to a quadratic function to model the response, as in Figure 2b. These results demonstrate that the linkage is capable of deterministic operation at nanometer and microradian scales, at least under some operating conditions and over some duration.

**Figure 6:**
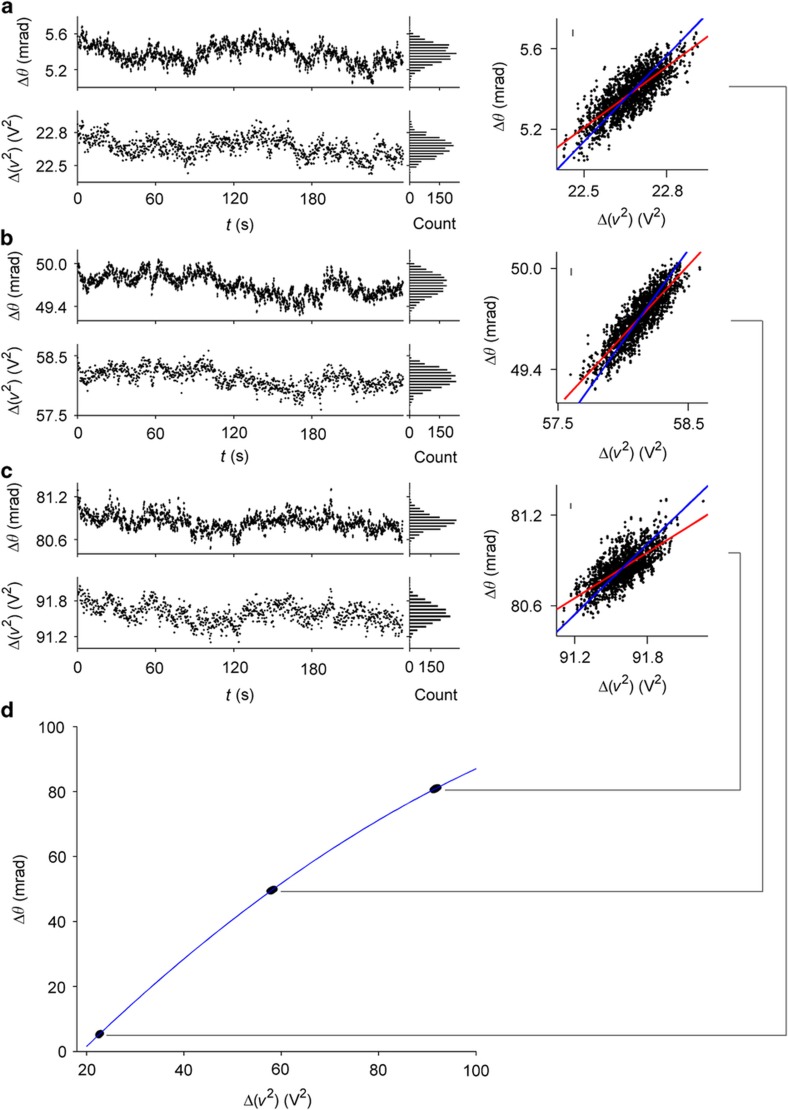
Input voltages with millivolt noise result in nondeterministic output. (**a–c**) Measurement series, histograms and scatterplots showing the local response of Δ*θ* to changes in Δ(*v*^2^) at nominal voltage values of 4.5 V, 7.5 V and 9.5 V, respectively. (**d**) Scatterplot showing the global response of Δ*θ* to changes in Δ(*v*^2^) with a fit to the response function as a blue line. Input voltages with millivolt noise result in nondeterministic coupling and decoupling of the linkage, with values of Δ*θ* scattered widely beyond measurement uncertainty. The scatterplots in (**a**–**c**) show linear fits to the local data around each nominal level of voltage as red lines, which have reduced slopes relative to the blue line. The vertical bars for values of Δ*θ* are empirical uncertainties that the main text describes. The vertical bars for values of Δ(*v*^2^) are uncertainties that the instrument manufacturer specifies. The insets of the small scatterplots in (**a**–**c**) show representative uncertainties for Δ*θ* in the upper left corners. The uncertainties for Δ(*v*^2^) are smaller than the data markers.

We then test the effects of extrinsic variation in input voltage by repeatedly actuating the linkage using input voltages with electrical noise at the millivolt scale, which is typical of power supplies for actuating microelectromechanical systems. The Supplementary Methods and Supplementary Figure S1 present details of this actuation protocol. The square wave amplitudes, Δ*v*, of the three levels of input voltage and associated values of root-mean-square noise are 4.5980 V and 0.0076 V, 7.5809 V and 0.0067 V, and 9.4763 V and 0.0075 V, respectively. Figures 6a–c show the corresponding values of Δ(*v*^2^) and Supplementary Figures S10b,d and f show the power spectral densities, which have the same frequency dependence as, but larger magnitude than, the inputs with submillivolt noise. Figures 6a–c show measurement series and histograms of the rotational output in response to electrical inputs with millivolt noise. Fluctuations in Δ*θ* positively correlate with fluctuations in Δ(*v*^2^), as the scatterplots of Δ*θ* versus Δ(*v*^2^) in Figures 6a–c show. Unlike the motion of the linkage in response to inputs with submillivolt noise, which is deterministic within measurement uncertainty, the motion of the linkage in response to input voltages with millivolt noise is nondeterministic. We quantify the variability of the rotational output by the s.d. of the residuals from the local linear fits. These values of 64 μrad, 71 μrad and 80 μrad are much larger than the measurement uncertainties of Δ*θ*, which are 16 μrad, 21 μrad and 18 μrad, respectively. These local fits to a linear function also manifest the fluctuating output as an apparent bias, systematically underestimating the slope relative to the global fit to a quadratic function, as the scatterplots in Figures 6a–c show. A previous report^35^ introduced this analysis for a linear response function over the range of Δ(*v*^2^) that Figure 6a shows and discussed the potential for biases that the present study elucidates. Noise in the input voltage degrades the precision of the rotational output and results in the nondeterministic operation of the microsystem. Because input noise on the millivolt scale is typical of power sources for actuating microelectromechanical systems, elucidating this effect is essential to optimizing their performance and reliability.

**Figure 7:**
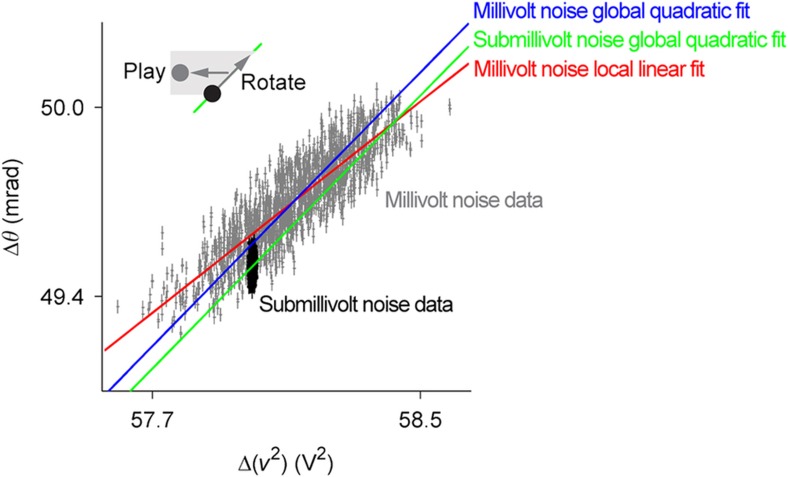
Asymmetric interaction between input noise and joint play degrades the rotational output of the microsystem. Inset: schematic illustrating the effects of noise on the coupling and decoupling of the links. Main panel: scatterplot comparing data and fits that show these effects. The black points are data from actuation of the linkage with an input voltage with submillivolt noise and the green line is the fit of the response function to the global set of data. The narrow scatter of the black points results primarily from measurement uncertainty. The gray points are data from actuation of the linkage with an input voltage with millivolt noise and the blue line is the fit of the response function to the global set of data. The red line is a fit of a linear function to only the local data with millivolt noise. The wide scatter of the gray points includes the effects of input noise and joint play, resulting in a measured value of Δ(*v*^2^) that is always less than or equal to the true value that produced the measured rotation of the rotating link. The effect of this scatter is evident as a shift from the green line to the blue line, and a reduced slope of the red line. The vertical bars are empirical uncertainties that the main text describes. The horizontal bars are the uncertainties specified by the instrument manufacturer.

Each transition of the linkage requires coupling of the translating link to the rotating link through the pin-in-slot joint. As Supplementary Figure S1 illustrates, the linkage remains nominally stationary in either a *low* or a *high* state between transitions of the input voltage. Such stationary operation is broadly relevant, because many applications of microelectromechanical systems do not require continuous motion, and in fact require the ability to maintain a stable position over an extended duration^10,28^. The *low* state of the linkage is, within uncertainty, independent of input noise, as Supplementary Table S3 shows. Therefore, the degraded output is a result of the effects of input noise on the *high* state of the linkage. A delay of ≈50 ms follows each transition from a *low* state to a *high* state, or vice versa. This delay precedes acquisition of the associated micrograph and measurement of the applied voltage, as Supplementary Figure S1 illustrates, and allows a spatiotemporal interaction between input noise and joint play. Immediately following each transition to the *high* state, at the start of this delay, the translating link and rotating link couple through sliding contact. During the delay, fluctuations in the input voltage due to noise produce corresponding fluctuations in the motion of the translating link. Fluctuations that increase Δ(*v*^2^) maintain sliding contact and coupling between the links, so that the translating link advances and rotates the rotating link. This interaction increases the measured values of both Δ*θ* and Δ(*v*^2^). In contrast, fluctuations that reduce Δ(*v*^2^) retract the translating link into the play in the joint, decoupling the links. In this way, the interaction between input noise and joint play results in a measured value of Δ(*v*^2^) that is always equal to or less than the true value that produced the measured value of Δ*θ*. This asymmetric interaction causes a nondeterministic output of the microsystem. Figure 7 shows a schematic of this concept and the corresponding results.

To clarify the effects of the interaction between input noise and joint play, Figure 7 directly compares the experimental results from similar nominal values of Δ(*v*^2^) for the cases of submillivolt and millivolt noise. The black points are measured data from actuation of the linkage with an input voltage with submillivolt noise and the green line is a quadratic function modeling the electrothermal response. The scatter in Δ*θ* of the black points is due primarily to measurement uncertainty. Within the uncertainty of the measurement, the input voltage with submillivolt noise maintains the coupling between the translating and rotating links and allows for a characterization of the electrothermal response of Δ*θ* to Δ(*v*^2^) without significant interaction of input noise and joint play. The gray points are measured data from actuation of the linkage with an input voltage with millivolt noise and the blue line is a quadratic function modeling the electrothermal response. The input noise causes fluctuations in the motion of the translating link while in the *high* state that decouple the translating and rotating links. The resulting data are asymmetrically scattered towards smaller values of Δ(*v*^2^), shifting the quadratic function. This shift is apparent as the offset between the blue and green lines in Figure 7. The interaction of input noise and joint play has a related effect on a linear fit to the data from a single input voltage with millivolt noise. Figure 7 shows this fit as a red line, which is both offset from the green line and has a reduced slope in comparison. The particular scatter of these data in Δ(*v*^2^) is the result of the temporal variation in the input noise at both low and high frequencies. The measured value of Δ*θ* corresponds to the maximum input voltage applied during the delay, whereas the reported value of Δ(*v*^2^) is the value measured at the end of the delay. These two values are correlated due to the pink noise of the input voltage, limiting the width of the scatter.

Electrical noise and mechanical play are common limitations of the operation of microelectromechanical systems that transfer motion through assemblies of parts. We find that their nondeterministic interaction causes the transfer of motion through our test system to become both quantitatively imprecise and qualitatively inconsistent. The resulting loss of performance and reliability at nanometer and microradian scales could be critical to practical applications requiring a precise and stable output.

## Conclusion

In this article, we report the first quantitative measurements of the transfer of motion through a microelectromechanical linkage at nanometer and microradian scales. We develop a novel combination of optical microscopy methods to track the motion of interacting parts in planar microsystems during operation. In our test system, we observe motion ranging from nearly ideal kinematics and deterministic transfer of motion to transient stiction and nondeterministic interaction of electrical noise and mechanical play. Our results emphasize, in several ways, the importance of precise measurements of complex microsystems. For example, at the current state of the art, theoretical models are unable to predict electrothermal actuation with accuracy equivalent to our measurement precision, which we will continue to improve. Furthermore, the practical nonidealities of fabrication tolerance, surface forces, electrical noise and their interactions can be difficult to control and model. Importantly, under ideal operating conditions, we find that microsystems with assemblies of moving parts in contact can have nearly ideal kinematics at nanometer and microradian scales, suggesting underexplored applications of such mechanisms. However, under nonideal but typical operating conditions, we find that even a simple assembly of microscale parts begins to show complex behavior at these scales. In a more complex system of many interacting parts in a more challenging operating environment, these various nonidealities could produce truly complex kinematics that would substantially alter the transfer of motion through an assembly of microscale parts in ways that would be difficult to predict. For this reason, this article opens the door to the experimental study and practical design of complex microsystems, with endless possibilities for applications. Such advances are necessary to realize the full potential of microsystems technology.
