# Supplementary material for: Transfer of motion through a microelectromechanical linkage at nanometer and microradian scales
Source: Microsyst Nanoeng. 2016 Sep 12;2:16055. doi: 10.1038/micronano.2016.55 (PMC5103322; doi:10.1038/micronano.2016.55)
Supplement: Supplementary Information [file micronano201655-s1.pdf]

Supplementary file

Transfer of motion through a microelectromechanical linkage at nanometer and microradian scales

Craig R. Copeland[1,2], Craig D. McGray[3,4], Jon Geist[3], Vladimir A. Aksyuk[1] and Samuel M. Stavis[1]

Microsystems & Nanoengineering (2016) **2,** 16055; doi:10.1038/micronano.2016.55; Published online: 12 September 2016

SUPPLEMENTARY METHODS

Linkage actuation

To measure the kinematics of the linkage, we step the input voltage v in nominal increments of 0.5 V from a nominal value of 0 V, up to a maximum value of approximately 9 V, and back down to 0 V, completing a single motion cycle. The reported value of v at each step is the mean value of four measurements from a digital multimeter. The root-mean-square noise of v is < 0.3 mV. We record 50 bright-field micrographs at each voltage step. For these measurements, we characterize the microsystem input in terms of the square of the input voltage v^2.

To test the repeatability of the rotational output, we vary v as a square wave at a frequency of 4 Hz and a duty cycle of 50% over 1 000 motion cycles. For these experiments, we characterize the microsystem input in terms of the change in the square of the input voltage, $\Delta(v^2) = v^2_{\text{high}}\ \ v^2_{\text{low}}$, between *high* and *low* transitions. We test several *high* values of v of several volts with root-mean-square noise of either several hundred microvolts or several millivolts. To obtain v with submillivolt noise, we use an arbitrary function generator to directly produce *high* values of $v \approx 4.6$ V, $v \approx 7.6$ V, and $v \approx 9.5$ V above a *low* value of $v \approx 0.2$ V. To obtain v with noise of several millivolts, we pass square waves with *high* values of $v \approx 46$ mV, $v \approx 76$ mV, and $v \approx 95$ mV above a *low* value of $v \approx 2$ mV from the arbitrary function generator through a 100× amplifier. The *low* value of v is the minimum offset of the amplified input voltage. We synchronize input voltage and fluorescence illumination to record fluorescence micrographs at the *low* and *high* values of v to track the sequential *low*-to-*high* and *high*-to-*low* transitions of the linkage, completing a single motion cycle. Figure S1 shows a timing diagram of this experiment. The duration of fluorescence illumination is 10 ms for each image. A digital multimeter records the mean input voltage over the first 2 ms of each 10 ms illumination pulse. Delays in electronic triggering of the multimeter limit the frequency of this measurement to below the maximum value of 60 Hz that the camera allows.

Nanoparticle deposition

We label the rotating link with a microscale constellation of fluorescent nanoparticles for imaging and tracking. The manufacturer specifies a nanoparticle size distribution with a mean value of 250 nm and a standard deviation (s.d.) of 9 nm, a fluorescence excitation peak at ≈630 nm, and a fluorescence emission peak at ≈645 nm. We dilute the nanoparticles in ethanol to a concentration of $\approx 10^8$ mL^{-1} and load this solution into a micropipette with an inner tip diameter of ≈5 μm. Brief contact of the micropipette with the rotating link deposits a microdroplet with a volume of ≈20 pL. Evaporation of the droplet leaves nanoparticles adsorbed to the surface.

Microscope system

For both bright-field and fluorescence microscopy, we mount the test system on an inverted optical microscope with a light emitting diode for illumination at ≈625 nm, an objective lens with a magnification of 50× and a numerical aperture of 0.55, and a complementary metal–oxide–semiconductor camera for recording micrographs. We use a 50/50 dichroic mirror for bright-field imaging and a set of fluorescence filters for fluorescence imaging. The resolution of the imaging system is ≈0.7 μm, so that individual nanoparticles appear as the point

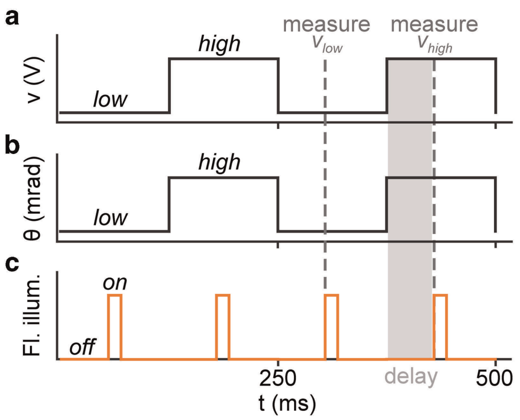

Figure S1 Linkage actuation and measurement timing for fluorescence microscopy. Schematic showing timing of the (**a**) square wave input voltage v, (**b**) resulting rotational output of the linkage θ, and (**c**) intensity of fluorescence illumination. We measure input voltages at 8 Hz and actuate the microsystem at the Nyquist frequency of 4 Hz. The dark gray dashed lines indicate measurements of input voltage. Illumination pulses excite the fluorescent nanoparticles labeling the rotating link, enabling measurement of θ following each transition of the linkage. The light gray box indicates the temporal delay between each transition of the linkage and the corresponding voltage measurement, allowing study of a spatiotemporal interaction between input noise and joint play.

[1]Center for Nanoscale Science and Technology, National Institute of Standards and Technology, Gaithersburg, MD 20899, USA; [2]Maryland Nanocenter, University of Maryland, College Park, MD 20742, USA; [3]Engineering Physics Division, National Institute of Standards and Technology, Gaithersburg, MD 20899, USA and [4]Modern Microsystems, Silver Spring, MD 20904, USA.
Correspondence: Samuel M. Stavis (samuel.stavis@nist.gov)

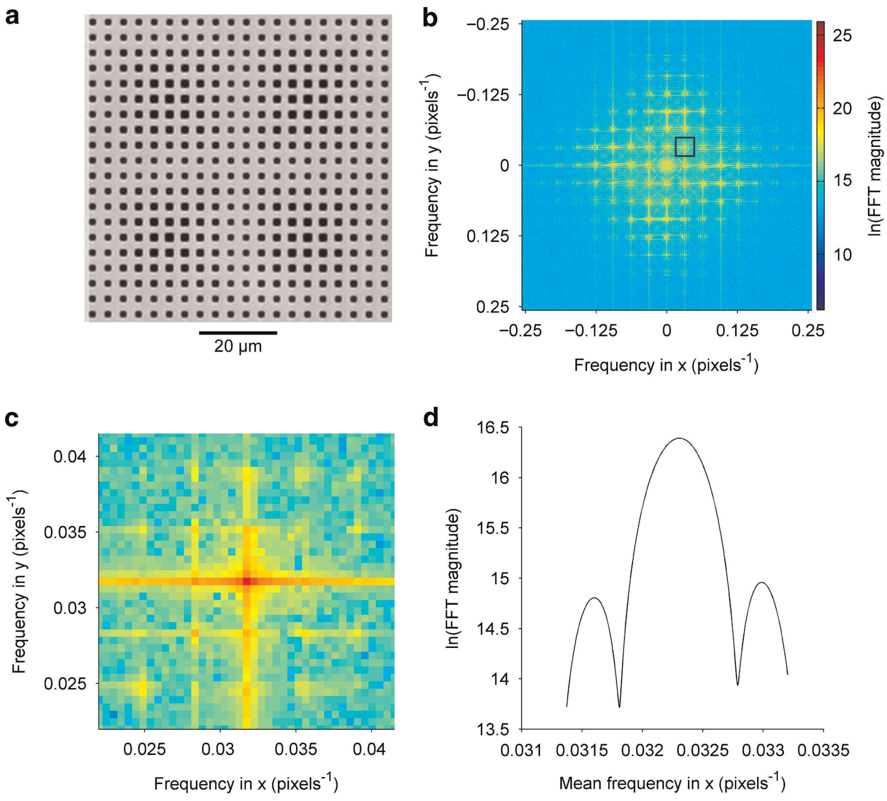

Figure S2 Magnification calibration. We record a bright-field micrograph of a test structure with a periodic pattern using the same optical microscope that we use for motion tracking. We analyze the pitch of the test structure with a discrete Fourier transform[1]. (**a**) Bright-field micrograph showing a representative region of the test structure, consisting of a square array of features with a pitch of 4 000 nm ± 2.5 nm. The latter value denotes a limit of uncertainty, which the manufacturer of the test structure specifies. The variable size of the features does not affect the Fourier spectrum in a way that influences this magnification calibration. (**b**) Fourier transform of the image in (a) using a standard discrete Fourier transform. We shift the spectrum to locate the zero-frequency component at the center and we scale the axes based on the image size of 2 048 pixels × 2 048 pixels. The black rectangle denotes a region of interest. (**c**) Magnified view of the region of interest in (**b**), corresponding to the fundamental frequency in the Fourier spectrum. The location of the frequency peak indicates the number of periodic features per pixel in the image of the test structure, with pixel resolution. (**d**) Plot showing the mean value in x of the fundamental frequency peak in (**b**) and (**c**) with denser sampling in frequency space using the Goertzel algorithm[2]. We take the mean value from all 2 048 rows of pixels in (**b**). The increased density of sampling around the fundamental frequency allows determination of the location of the frequency peak with higher resolution than a standard discrete Fourier transform. In this way, we determine that the spatial frequency of the features in the image for both the x and y directions is 0.0318 pixels^{-1}. The image pixel size is then 0.0318 pixels^{-1}· (4 000 nm ± 2.5 nm) = 127.2 nm per pixel ± 0.1 nm per pixel, with the latter value representing a conservative estimate of the limit of uncertainty.

spread function of the imaging system. The imaging system begins to resolve the linkage pin, which has a diameter of ≈ 1 μm, and further resolves the etch holes, which have a diameter of ≈ 2 μm.

Supplementary note 1 Uncertainty evaluation for bright-field microscopy

The values of D are displacements between the mean positions of the linkage pin from a set of 50 images of the nominally static linkage at each voltage step with respect to $v = 0$ V. The values of θ are the rotations between the mean positions of the etch holes from measurements of the set of 50 images of the nominally static linkage at each voltage step with respect to $v = 0$ V. The uncertainties are the sum in quadrature of the root-mean-square displacements or rotations from the set of 50 images at each voltage step and the $v = 0$ V voltage step, divided by $\sqrt{2}$.

Supplementary note 2 Derivation of the kinematic model

We derive a kinematic model for the coupled links in sliding contact, beginning with the motion of the linkage pin relative to the pivot. This model describes the hypothetical kinematics of the linkage for sliding contact along the center line of the rotating link, in the absence of play in the joint. With reference to Figures 3b and 3c, the law of sines gives:

$$\sin\theta_{\vec{r}} = \frac{D\sin(\pi \quad \Lambda_0)}{r(D)} \tag{S1}$$

Where $r(D)$ is the distance between the linkage pin and the pivot as a function of the distance D that the translating link moves. The law of cosines gives:

$$\cos\theta_{\vec{r}} = \frac{r_0^2 + r(D)^2 \quad D^2}{2r_0 r(\mathrm{D})} \tag{S2}$$

Using (S1) to eliminate $r(D)$ in (S2) and solving for $\theta_{\vec{r}}$ gives:

$$\theta_{\vec{r}}(D) = \frac{1}{2}\left(\Lambda_0 \quad \mathrm{Real}\left[i\cdot\ln\left(\frac{r_0 + De^{i\Lambda_0}}{D + r_0e^{i\Lambda_0}}\right)\right]\right) \tag{S3}$$

We derive a model for the angular play in the joint, or the maximum rotation of the rotating link while it is decoupled from

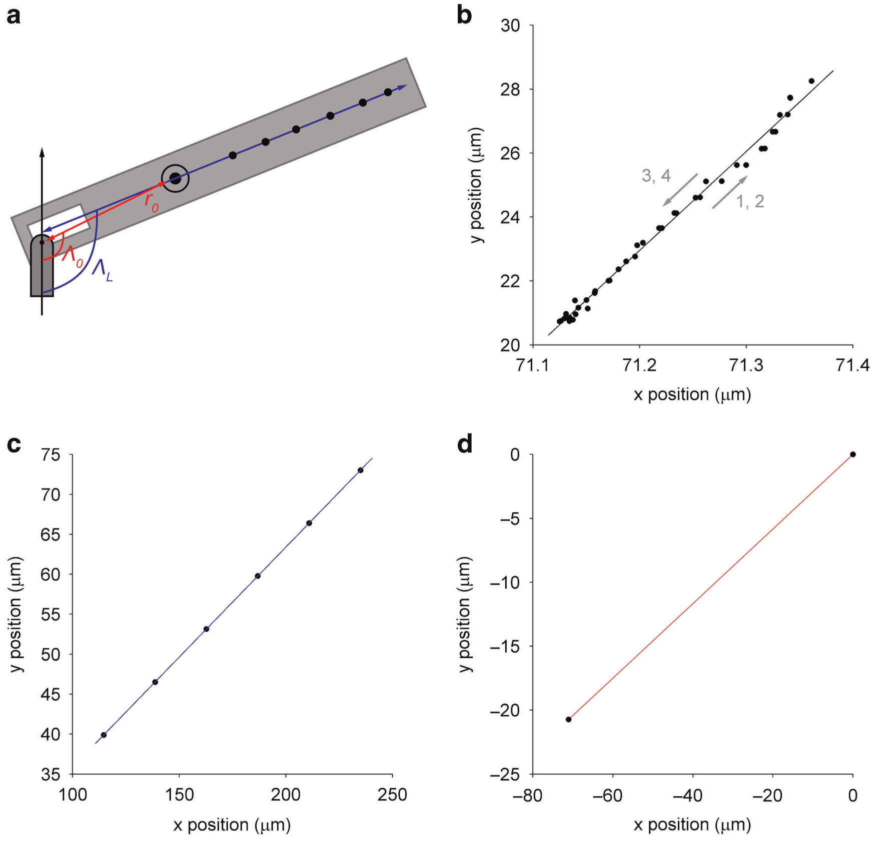

Figure S3 Superresolution measurement of Λ_0 and Λ_L. (**a**) Schematic of the linkage showing two angles, Λ_0 and Λ_L, determining the kinematics in Supplementary Note 2. (**b**) Scatterplot showing a linear fit of the trajectory of the linkage pin on the translating link, giving a slope of 30.95 ± 0.43. (**c**) Scatter plot showing a linear fit of the centroid positions of six etch holes on the rotating link at $v = 0$ V, giving a slope of 0.27517 ± 0.000087. (**d**) Scatterplot showing a linear fit between the positions of the linkage pin and the pivot at $v = 0$ V, giving the slope of $\overrightarrow{r}_0$ as 0.29136 ± 0.000096. The relation $\tan(\pi \quad \Lambda) = \frac{m_1 \quad m_2}{1+m_1 m_2}$ between two lines with slopes m_1 and m_2 determines the angle Λ between the lines. In combination with the values from (**a**–**d**), this analysis gives $\Lambda_0 = 1.8866$ rad ± 0.0005 rad and $\Lambda_L = 1.8731$ rad ± 0.0014 rad.

the translating link. With reference to Figure 3d, the law of cosines gives:

$$\theta_{play}(D) = \cos^{\ 1}\left(1 \quad \frac{w^2}{2 \cdot r(D)^2}\right) \qquad (S4)$$

where the width of the slot $w = D^0_{\text{play}} \sin(\pi \quad \Lambda_L)$. Using the identity $\sin^2\theta + \cos^2\theta = 1$, in combination with Equations (S1) and (S2) gives:

$$r(D) = \left(\frac{D^4 + r_0^4 \quad 2D^2 r_0^2 + 4D^2 r_0^2 \sin^2\Lambda_0}{D^2 + r_0^2 + 2Dr_0[1 \quad \sin^2\Lambda_0]^{1/2}}\right)^{1/2} \qquad (S5)$$

Equations (2) and (4) in the main text then follow from Figures 3b-d.

Supplementary note 3 Uncertainty evaluation for fluorescence microscopy

In previous studies we introduced the use of a constellation of fluorescent nanoparticles for tracking the motion of a microscale mechanical body[3–5]. In the present study we advance this measurement method through a novel analysis of experimental uncertainties, proceeding in the following order. Initial measurements of the motion of a nominally static constellation of nanoparticles in the absence of actuation of the linkage quantify the uncertainty associated with the measurement system. Subsequent analysis of the nominally static constellation in the *low* state between repeated motion cycles determines the specific value of this uncertainty for each experiment. Finally, correction of errors due to systematic deformation of the constellation resulting from motion of the rotating link out of the image plane ensures accuracy. This analysis enables quantitative tests of the performance and reliability of the microelectromechanical linkage at nanometer and microradian scales. This allows for a meaningful assessment of the output of the microsystem as deterministic or nondeterministic at length and angle scales which previous studies have not investigated.

Sources of uncertainty in the measurements of translation and rotation include apparent motion due to photon shot noise and variation in the optoelectronic properties of individual pixels[6], as well as actual motion due to vibration of the linkage, and vibration and drift of the microscope system. We quantify the total uncertainty due to the measurement system by analyzing the measured positions and orientations of the constellation in the absence of actuation of the linkage. Figures S6a–c show the motion in x, y and θ of the nominally static and rigid nanoparticle constellation over 250 s, and Figures S6d–f show the corresponding Allan deviations[7].

Drift of the microscope stage results in systematic translations of the nanoparticle constellation in x and y over the full duration of each experiment. In contrast, the mean orientation θ of the constellation is nearly constant in time and fluctuations of θ are normally distributed, which is consistent with the microscope stage lacking a rotational degree of freedom. This useful insensitivity of rotation to a common source of error in optical microscopy allows for direct and quantitative comparison of

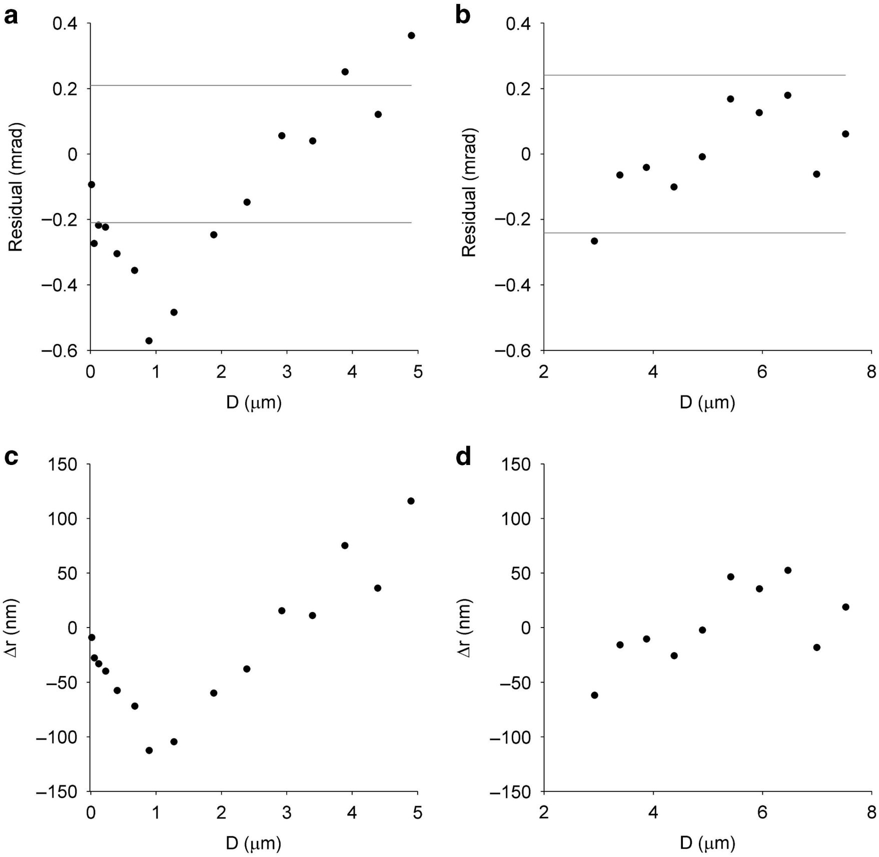

Figure S4 Comparison of experimental and theoretical values of $\theta(D)$. Scatterplots showing the residuals between experimental and model values for (**a**) section 4 of the hysteresis loop and (**b**) section 2 of the hysteresis loop in Figure 3d. Systematic deviations from the kinematic model can become significant beyond a confidence interval of 95%. The mean value of the relative residuals is $\approx 5\%$. Gray lines are at 2 s.d. These deviations are consistent with shifts of the point of rotation that change the magnitude of $\vec{r}$ by Δr. (**c**), (**d**) Scatterplots showing the values of Δr that minimize the systematic deviations in (**a**), (**b**). These empirical adjustments of $\vec{r}$ reduce the deviations to < 1 μrad.

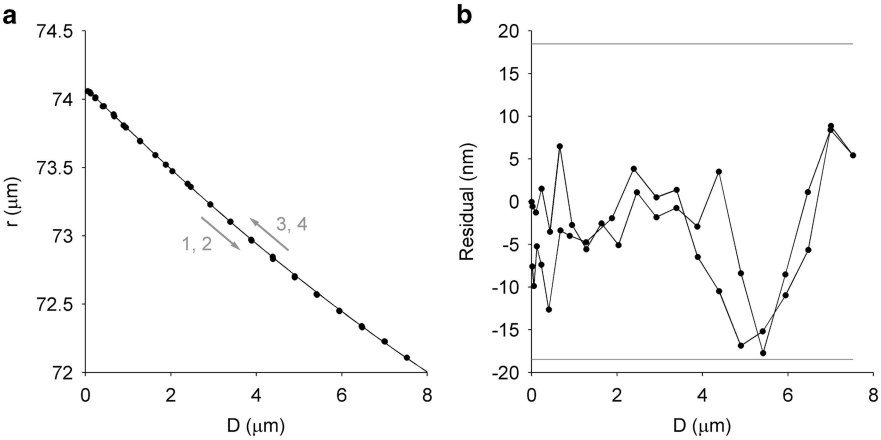

Figure S5 Comparison of experimental and theoretical values of $r(D)$. (**a**) Scatterplot showing the measured values of $r(D)$ and predicted values of $r(D)$ from Equation (S2) are in apparent agreement. The uncertainties are smaller than the data markers. (**b**) Scatterplot showing the residuals from (**a**), which show that the trajectory of the translating link is not perfectly straight, producing a systematic deviation that is barely within a confidence interval of 95%. Figure S3 also shows the irregular trajectory of the translation link. Gray lines are at 2 s.d.

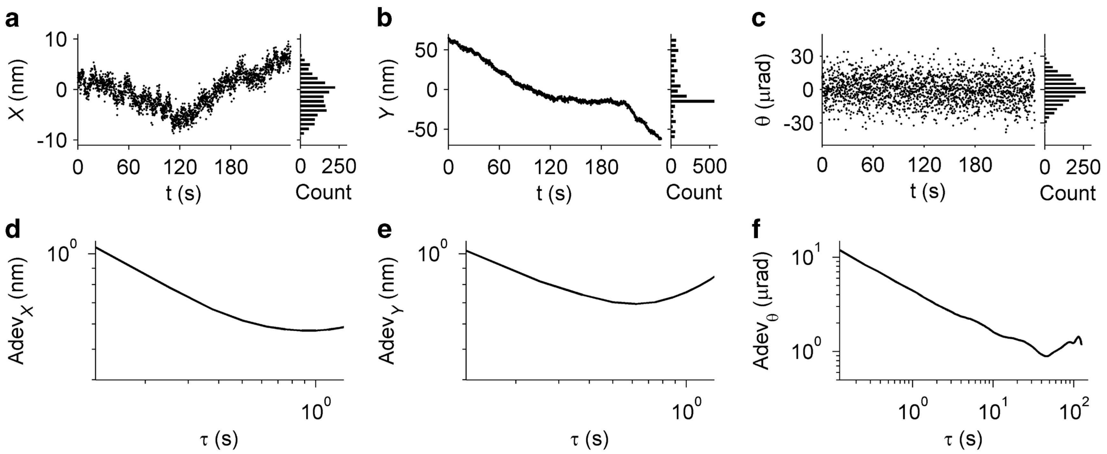

Figure S6 Representative uncertainties from the measurement system. (**a-c**) Scatterplots and histograms showing the motion of the nanoparticle constellation in the absence of actuation of the linkage. The constellation translates systematically in x and y. In contrast, the orientation θ of the constellation fluctuates randomly. (**d-f**) Plots showing Allan deviation for *X*, *Y* and θ. The vertical axis intercepts at $\tau = 0.125$ s give the uncertainties of single measurements. The uncertainties for measurements of motion are a factor of $\sqrt{2}$ greater than these Allan deviations and are equivalent to the root-mean-square displacement in *x*, *y* and θ. This figure and Figure S7 show that measurements of θ are insensitive to microscope drift.

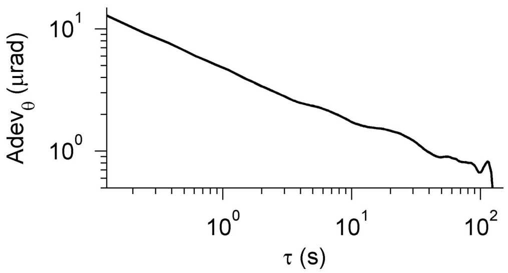

Figure S7 Allan deviation of rotation in the absence of non-ideal indicators. Plot showing that the measurement of the orientation of the constellation is insensitive to microscope drift, as is evident by a minimum value of the Allan deviation for θ occurring at the maximum possible value of τ. Temporal averaging of white noise gives a minimum Allan deviation for θ of 0.47 µrad.

θ throughout the time series, as well as temporal averaging of multiple measurements to reduce the effects of white noise.

The Allan deviation of measurements of the orientation of the constellation θ is $ADev_\theta(\tau) = \sqrt{\frac{1}{2}\langle(\theta_{k+1} \quad \theta_k)^2\rangle_\tau}$, with isomorphic expressions for *X* and *Y*. The value θ_k is the mean value of θ over an interval of duration τ, while *k* indexes the interval in the time series. The index *k+1* advances the interval through the time series, setting the maximum value of τ to half the number of images. Additionally, taking the difference between sliding mean values reduces the statistical error, where the index *k+1* advances the interval by a single measurement through the time series. Random translations and rotations occur on the time scale of single measurements due to vibration and photon shot noise. The minimum averaging interval of the Alan deviation $\tau = 0.125$ s gives the corresponding uncertainty of a single measurement of position or orientation[7], and is equivalent to the root-mean-square displacement or rotation divided by $\sqrt{2}$. Allan deviations for sliding intervals with increasing values of τ provide information about the limits of the measurement over a duration extending beyond the time scale of a single motion cycle. Figures S6 shows that, for all *X*, *Y* and θ, the Allan deviation follows the relationship of $ADev \propto \tau^{\ \frac{1}{2}}$, corresponding to averaging of white noise, until reaching a minimum value at $\tau \approx 1$ s for motion in x and y, and $\tau \approx 50$ s for motion in θ. The point of minimum Allan deviation indicates, for x and y, the transition from a measurement limited by random noise to a measurement limited by systematic drift. In contrast, for θ, asymmetric variation of the intensity profile of a small number of aggregate particles in an etch hole limit the minimum value of the Allan deviation. For all *X*, *Y* and θ, the minima of the Allan deviations quantify the minimum values of the empirical uncertainties that are achievable by reducing the bandwidth of the measurement while holding constant all other experimental parameters. For Figures S6, these values are 0.57 nm for *X*, 0.69 nm for *Y* and 0.89 µrad for θ. For a particle constellation of individual nanoparticles, without any non-ideal indicators, the Allan deviation of θ continually decreases with increasing τ, confirming the absence of rotational drift. Figure S7 shows this result, which further decreases the minimum empirical uncertainty of θ that is achievable through temporal averaging to 0.47 µrad.

In a previous study we derived expressions of centroid precision and orientation precision[4], which are the minimum uncertainties of localizing and orienting a constellation of point sources of variable brightness on a rigid body in the image plane of a microscope. Centroid precision and orientation precision result from photon shot noise, image pixilation, and background noise. The latter is negligible for the experimental measurements in the present study. Table S1 presents these values of minimum uncertainty for single motion cycles, which are useful metrics for analysis of additional sources of experimental or computational uncertainty. The experimental uncertainties are greater than the fundamental physical limits, for the parameters of this particular measurement, by a factor of only 1.3 to 1.6. The experimental values exceed the minimum uncertainty in part due to localization errors from a few non-ideal indicators consisting of small aggregates of subresolution nanoparticles in etch holes. Inclusion of these indicators in the analysis reduces the empirical measurement uncertainty by increasing the number of photons detected, but with an efficiency below that of photons from individual particles.

Measurements of motion between sequential images have uncertainties that are greater by a factor of $\sqrt{2}$, equivalent to the

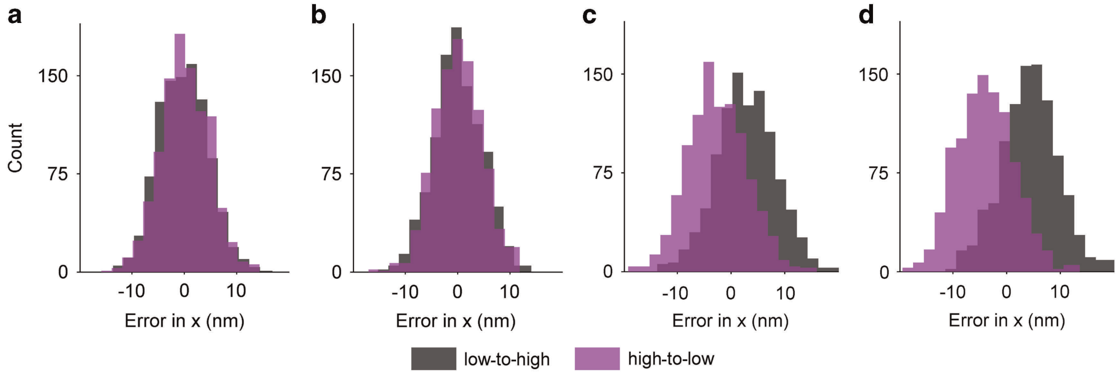

Figure S8 Biases in the relative position error of a representative nanoparticle due to motion of the rotating link out of the image plane. (**a**–**d**) Histograms showing errors in the x position of a representative nanoparticle for actuation of the linkage with input voltages of (**a**) 0 V, (**b**) 4.6 V, (**c**) 7.6 V, and (**d**) 9.5 V. For actuation with 7.6 V and 9.5 V, in particular, systematic deformation of the constellation causes the mean error for transitions between the *low* and *high* states to be biased and nonzero.

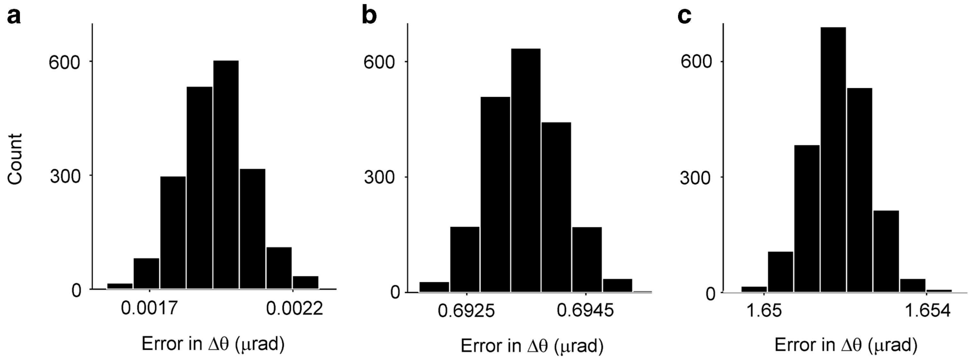

Figure S9 Errors in measured rotations due to deformation of the nanoparticle constellation. (**a**-**c**) Histograms showing errors in the measured rotations of the rotating link for actuation with (**a**) 4.6 V, (**b**) 7.6 V, and (**c**) 9.5 V. Correction of systematic biases in the position of each particle eliminates these errors.

Table S1 Comparison of empirical and minimum uncertainties

	Empirical uncertainty	Minimum uncertainty	Ratio of empirical to minimum uncertainties
X	1.1 nm	0.68 nm	1.55
Y	1.0 nm	0.68 nm	1.50
θ	12 µrad	9.3 µrad	1.28

Table S2 Rotational variation for actuation with input voltages with submillivolt noise

Input voltage (V)	RMS rotation (µrad)		
	Low-low*	High-high*	Low-high*
**0**	17	16	17
**4.6**	18	17	18
**7.6**	22	22	23
**9.5**	23	21	22

*These values are free of input noise, so that the variation is due only to measurement uncertainty.

Table S3 Rotational variation for actuation with input voltages with millivolt noise

Input voltage (V)	RMS rotation (µrad)		
	Low-low	High-high*	Low-high*
**4.6**	16	59	64
**7.6**	21	69	71
**9.5**	18	85	80

*These values are larger than measurement uncertainty due to fluctuations resulting from noise in the input voltage.

Table S4 Transformation errors for transitions from *low* to *high* increase with input voltage

Input voltage (V)	RMS error in x (nm)			RMS error in y (nm)		
	Low-low	High-high	Low-high	Low-low	High-high	Low-high
**4.6**	4.2	4.2	4.3	4.1	4.1	4.2
**7.6**	5.3	5.4	5.8*	5.0	5.1	5.3
**9.5**	4.9	4.9	5.8*	5.2	4.8	5.2

*Errors for transitions between *low* and *high* are greater than errors for transitions between *low* and *low* and between *high* and *high*.

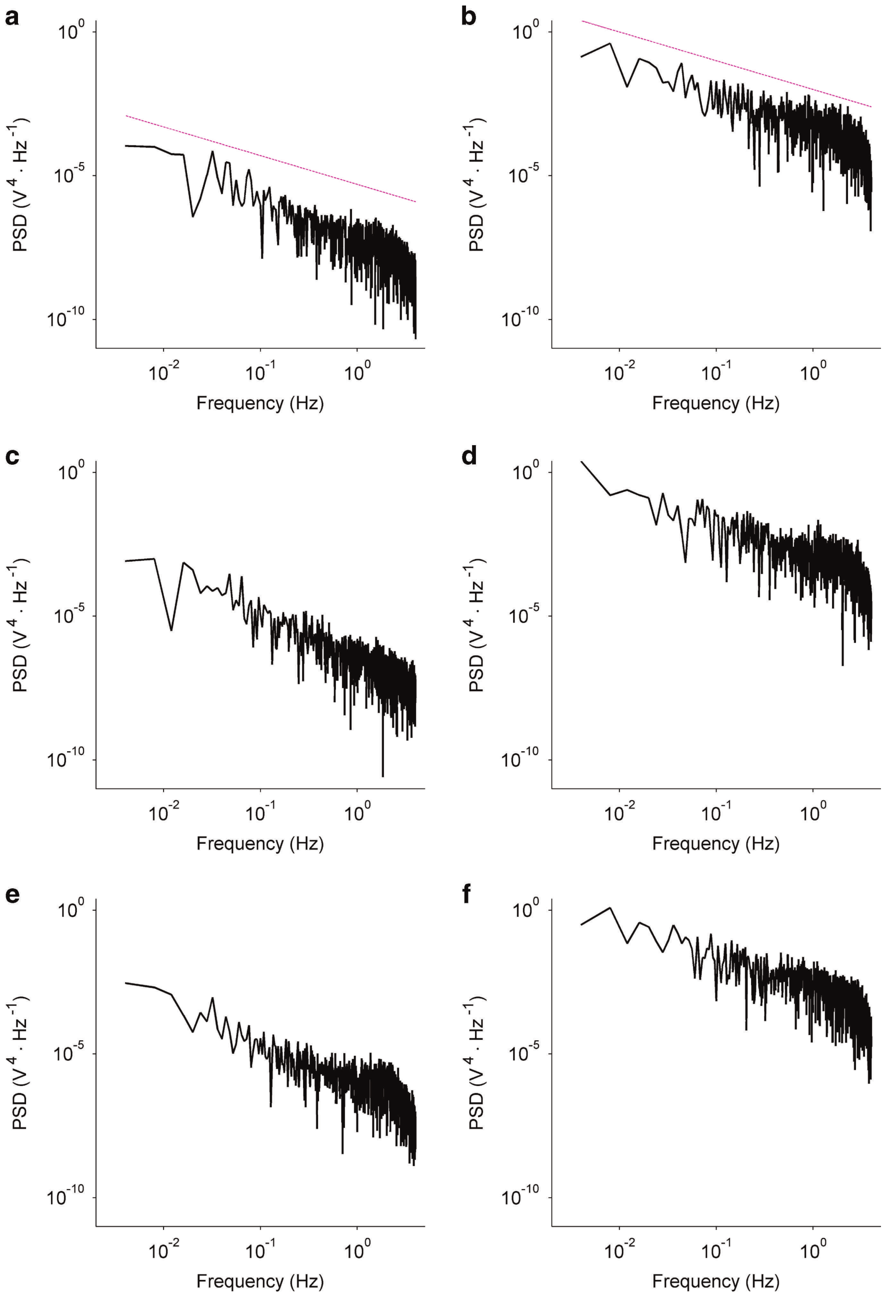

Figure S10 Power spectral densities of $\Delta(v^2)$. Plots showing the power spectral density of the (**a**) 4.6 V input with submillivolt noise, (**b**) 4.6 V input with millivolt noise, (**c**) 7.6 V input with submillivolt noise, (**d**) 7.6 V input with millivolt noise, (**e**) 9.5 V input with submillivolt noise, and (**f**) 9.5 V input with millivolt noise. The power spectral densities show a frequency dependence that is generally consistent with pink noise. The offset pink lines in (**a**) and (**b**) have a slope of -1 for reference. The absence of any other salient features in the spectral densities indicates that any input noise at higher frequencies, which is present in the spectral densities from aliasing, follows a similar dependence.

Table S5 Rotational variation and transformation errors after correction of systematic biases

	RMS rotation (μrad)			RMS error in x (nm)			RMS error in y (nm)		
Input voltage (V)	Low-low	High-high	Low-high	Low-low	High-high	Low-high	Low-low	High-high	Low-high
**4.6**	18	17	18	4.2	4.2	4.2	4.1	4.1	4.1
**7.6**	22	22	23	5.3	5.4	5.3*	5.0	5.1	5.1
**9.5**	23	21	22	4.9	4.9	4.9*	5.2	4.8	4.9

*Correction of biases for transitions between *low* and *high* states reduces the excess error caused by deformation of the constellation

root-mean-square displacement or rotation. Representative experimental uncertainties are 1.4 nm for displacements in x and y, and 17 μrad for rotations in θ. These uncertainties fluctuate by as much as 20%, requiring measurement during actuation of the linkage to quantify. The *low* states of the rotating link are insensitive to noise in the input and nominally identical, so that the root-mean-square deviation of the rotations of the rotating link while in this nominally static state give the uncertainty of rotation measurements for each level of input voltage. Table S2 shows these values for transitions of the rotating link between the *low* states of sequential motion cycles, the *high* states of sequential motion cycles, and between the *low* and *high* states of each motion cycle for actuation with input voltages with submillivolt noise. The root-mean-square rotations at each level of voltage are equivalent for all three transitions, indicating that the variation does not change with the motion of the linkage and must therefore result only from measurement uncertainty. We conservatively select the largest root-mean-square rotation from the three nominally static states as the measurement uncertainty of rotation for each voltage level.

Actuation of the linkage with input voltages with millivolt noise causes significant variation in the rotational output of the linkage. Therefore, in this case, only the root-mean-square rotation for transitions of the rotating link between the *low* states of sequential motion cycles inform of measurement uncertainty. Table S3 shows these values.

The preceding analysis determines the uncertainty due only to the measurement system. The actuation of the linkage could result in additional error from apparent deformation of the nanoparticle constellation, due to, for example, motion of the rotating link out of the image plane. Such deformation would produce relative motion of corresponding particles in the constellation for transitions between the *low* and *high* states of the linkage that is inconsistent with a rigid transformation. We quantify this deformation by changes in the root-mean-square error of the rigid transformation. Systematic deformation of the constellation between *low* and *high* states produces a repeatable bias in the position of each particle, increasing the error of the transformations by up to 25% for actuation with input voltages of 7.6 and 9.5 V. The root-mean-square differences in the position of corresponding particles quantify the error in the transformation from image *i* to image *j* that define the motion of the rotating link. In the case that the two images *i* and *j* do not bound a transition of the linkage between the *low* and *high* state, as in the absence of actuation of the linkage or for transitions between the *low* or *high* states of sequential motion cycles, the error results only from shot noise and variable pixel characteristics. In the case where the images *i* and *j* bound a transition between the *low* and the *high* states, any motion of the rotating link out of the image plane produces additional error due to deformation of the constellation. Table S4 shows these errors for the three nominal levels of input voltage, as well as in the absence of actuation of the linkage. The error increases by approximately 10% and 25% in the *x* direction as a result of actuation of the linkage with input voltages of 7.6 and 9.5 V, respectively. This systematic deformation of the constellation between the *low* and *high* states causes repeatable errors in the estimates of the position of each particle. These errors are equal and opposite for transitions from *low* to *high* and *high* to *low,* as Figure S8 shows for a representative nanoparticle.

Correction of these biases removes the increased error in the transformation of the constellation but does not change the variation in the measurements of rotation, showing that the deformation of the constellation is repeatable within measurement uncertainty. Table S5 shows these values. Without correction, the biases in the position of each particle result in a small error in the measurements of rotation that is on the order of 0.001%, as Figure S9 shows. Such errors from motion out of the image plane can be much larger for nominally planar microsystems, however, as we will show in a future study.

REFERENCES

1 Dai X, Xie H, Li C *et al.* High-accuracy magnification calibration for a microscope based on an improved discrete Fourier transform. *Optical Engineering* 2013; **52**: 114102–114102.

2 Goertzel G.. An algorithm for the evaluation of finite trigonometric series. *The American Mathematical Monthly* 1958; **65**: 34–35.

3 Copeland CR, McGray CD, Geist J *et al.* Characterization of electrothermal actuation with nanometer and microradian precision. 18th International Conference on Solid-State Sensors, Actuators and Microsystems (TRANSDUCERS 2015); 21-25 Jun 2015; Anchorage, AK, USA; 2015: 792–795.

4 McGray C, Copeland CR, Stavis SM *et al.* Centroid precision and orientation precision of planar localization microscopy. *Journal of Microscopy*, 2016 2016.

5 McGray CD, Stavis SM, Giltinan J *et al.* MEMS Kinematics by super-resolution fluorescence microscopy. *Journal of Microelectromechanical Systems* 2013; **22**: 115–123.

6 Huang F, Hartwich TMP, Rivera-Molina FE *et al.* Video-rate nanoscopy using sCMOS camera-specific single-molecule localization algorithms. *Nature Methods* 2013; **10**: 653–658.

7 Czerwinski F, Richardson AC, Oddershede LB.. Quantifying noise in optical tweezers by allan variance. *Optics Express* 2009; **17**: 13255–13269.
